# The Human Host‐Defense Peptide Cathelicidin LL‐37 is a Nanomolar Inhibitor of Amyloid Self‐Assembly of Islet Amyloid Polypeptide (IAPP)

**DOI:** 10.1002/anie.202000148

**Published:** 2020-04-30

**Authors:** Valentina Armiento, Kathleen Hille, Denise Naltsas, Jennifer S. Lin, Annelise E. Barron, Aphrodite Kapurniotu

**Affiliations:** ^1^ Division of Peptide Biochemistry TUM School of Life Sciences Emil-Erlenmeyer-Forum 5 85354 Freising Germany; ^2^ Department of Bioengineering Stanford University 443 Via Ortega, Shriram Center for Bioengineering Stanford CA 94305 USA

**Keywords:** amyloids, inhibitors, protein interactions, self-assembly, type 2 diabetes

## Abstract

Amyloid self‐assembly of islet amyloid polypeptide (IAPP) is linked to pancreatic inflammation, β‐cell degeneration, and the pathogenesis of type 2 diabetes (T2D). The multifunctional host‐defence peptides (HDPs) cathelicidins play crucial roles in inflammation. Here, we show that the antimicrobial and immunomodulatory polypeptide human cathelicidin LL‐37 binds IAPP with nanomolar affinity and effectively suppresses its amyloid self‐assembly and related pancreatic β‐cell damage in vitro. In addition, we identify key LL‐37 segments that mediate its interaction with IAPP. Our results suggest a possible protective role for LL‐37 in T2D pathogenesis and offer a molecular basis for the design of LL‐37‐derived peptides that combine antimicrobial, immunomodulatory, and T2D‐related anti‐amyloid functions as promising candidates for multifunctional drugs.

## Graphical abstract

**One for all**: A high‐affinity amyloid‐suppressing interaction between the human antimicrobial and immunomodulatory polypeptide cathelicidin LL‐37 and the key type 2 diabetes (T2D) amyloid polypeptide IAPP was identified. The results suggest a protective role for LL‐37 in T2D pathogenesis and offer a molecular basis for the design of novel molecules combining antimicrobial, immunomodulatory, and anti‐amyloid functions as multifunctional drug candidates.

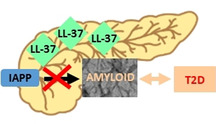

Amyloid self‐assembly of islet amyloid polypeptide (IAPP) is linked to pancreatic β‐cell degeneration and the pathogenesis of type 2 diabetes (T2D).^1^ The 37‐residue IAPP is secreted from the β‐cells together with insulin and acts in its soluble form as a neuropeptide regulator of glucose homeostasis (Scheme 1).^1^ However, under conditions of T2D, the intrinsically disordered but highly amyloidogenic IAPP self‐assembles into cytotoxic oligomers and amyloid fibrils, which mediate pancreatic inflammation and β‐cell degeneration.^1^, ^2^

**Scheme 1:**
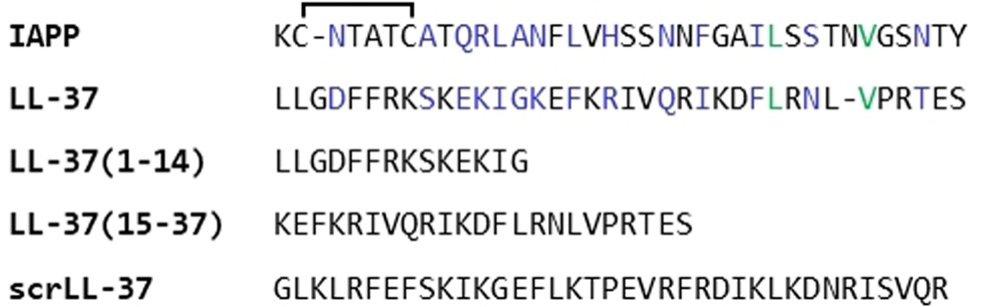
Primary structures of IAPP, LL‐37, scrambled LL‐37 (scrLL‐37), and LL‐37 segments synthesized and studied (IAPP has a C‐terminal amide; LL‐37 and related peptides have a C‐terminal COOH). IAPP and LL‐37 sequence alignment was performed by LALIGN;^8^ similar residues are blue and identical green coloured.

The multifunctional host‐defense peptides (HDPs) cathelicidins play crucial roles in inflammatory processes, including both pro‐ and anti‐inflammatory ones.^3^ So far, the only known human cathelicidin is LL‐37 (Scheme 1).3b LL‐37 is a 37‐residue polypeptide that is broadly expressed by a plethora of immune and non‐immune cells, including the β‐cells of pancreas.3a, 3e, 4 LL‐37 plays a crucial role in innate immunity; its best known functions are its broad‐spectrum antimicrobial activity and its potent immunomodulatory effects.^3^ Importantly, secretion of the mouse LL‐37 orthologue cathelicidin related antimicrobial peptide (CRAMP) by pancreatic β‐cells was recently found to suppress pancreatic β‐cell inflammation in a mouse model of type 1 diabetes (T1D) by converting inflammatory cells into regulatory ones.^4^ In addition, CRAMP/LL‐37 treatment promoted insulin and glucagon secretion and enhanced islet function.4b Thus, a protective role for LL‐37 in T1D has been suggested.^4^ The multifunctional nature of LL‐37 makes it of high biomedical importance and numerous studies toward the design of LL‐37‐derived peptides with antimicrobial or immunomodulatory functions have been reported.^3^, ^5^

Increasing evidence suggests that interactions of amyloidogenic polypeptides with other polypeptides are crucial modulators of amyloid self‐assembly.^6^ For instance, high‐affinity interactions of non‐fibrillar species of IAPP with insulin or amyloid β peptide (Aβ40(42)) of Alzheimer's disease (AD) have been found to suppress IAPP amyloidogenesis in vitro.6c, 6e, 7 In addition, LL‐37 was recently shown to interact with Aβ42 resulting in suppression of Aβ42 amyloidogenesis and neuroinflammation in vitro.6b

Based on the above information and in particular on the presence of LL‐37 in the pancreas, we asked whether it might also interact with IAPP. Notably, LL‐37 and IAPP share a remarkable (42 %) sequence similarity (Scheme 1). Herein, we show that LL‐37 in fact binds with nanomolar affinity to IAPP and effectively suppresses its amyloid self‐assembly and related pancreatic β‐cell‐damage in vitro. In addition, we identify key LL‐37 segments that mediate its interaction with IAPP.

We first addressed the question of whether LL‐37 might interfere with IAPP amyloidogenesis and the formation of cell‐damaging assemblies by using the ThT binding assay in combination with TEM and a cell viability assay (Figure 1). In fact, LL‐37 (1:1 relative to IAPP) effectively suppressed IAPP amyloid self‐assembly (Figure 1 a). The results of the ThT assay were confirmed by TEM, which revealed amorphous aggregates as major species in aged IAPP‐LL‐37 mixtures (Figure 1 b). Interestingly, a few fibrils were also observed in aged LL‐37 alone in addition to amorphous aggregates consistent with previous findings.^9^ The dose‐dependence of the amyloid inhibitory effect was confirmed by additional studies (Figure S1). Addition of the above solutions to cultured pancreatic β‐cells (RIN5fm) and determination of cell damage through a the 3‐[4,5‐dimethylthiazol‐2‐yl]‐2,5‐diphenyltetrazolium bromide (MTT) reduction assay showed that LL‐37 effectively suppressed formation of cytotoxic IAPP assemblies as well (Figure 1 c,d). Of note, scrambled LL‐37 (scrLL‐37) was unable to inhibit up to an at least 10‐fold molar excess and LL‐37 alone was not cytotoxic (Scheme 1, Figures 1 a–c & S2). To quantify the inhibitory activity of LL‐37, titrations of cytotoxic IAPP with LL‐37 were performed and an IC_50_ of 17(±1.7) nm was obtained (Figure 1 d); thus, LL‐37 is a nanomolar inhibitor of IAPP cytotoxic self‐assembly. Furthermore, we asked whether LL‐37 may also interfere with nucleation of IAPP fibrillogenesis by addition of seed amounts of preformed IAPP fibrils (fIAPP). In fact, in the presence of LL‐37 (1/1), the seeding effect of fIAPP (10 %) was fully suppressed (Figure 1 e).

**Figure 1:**
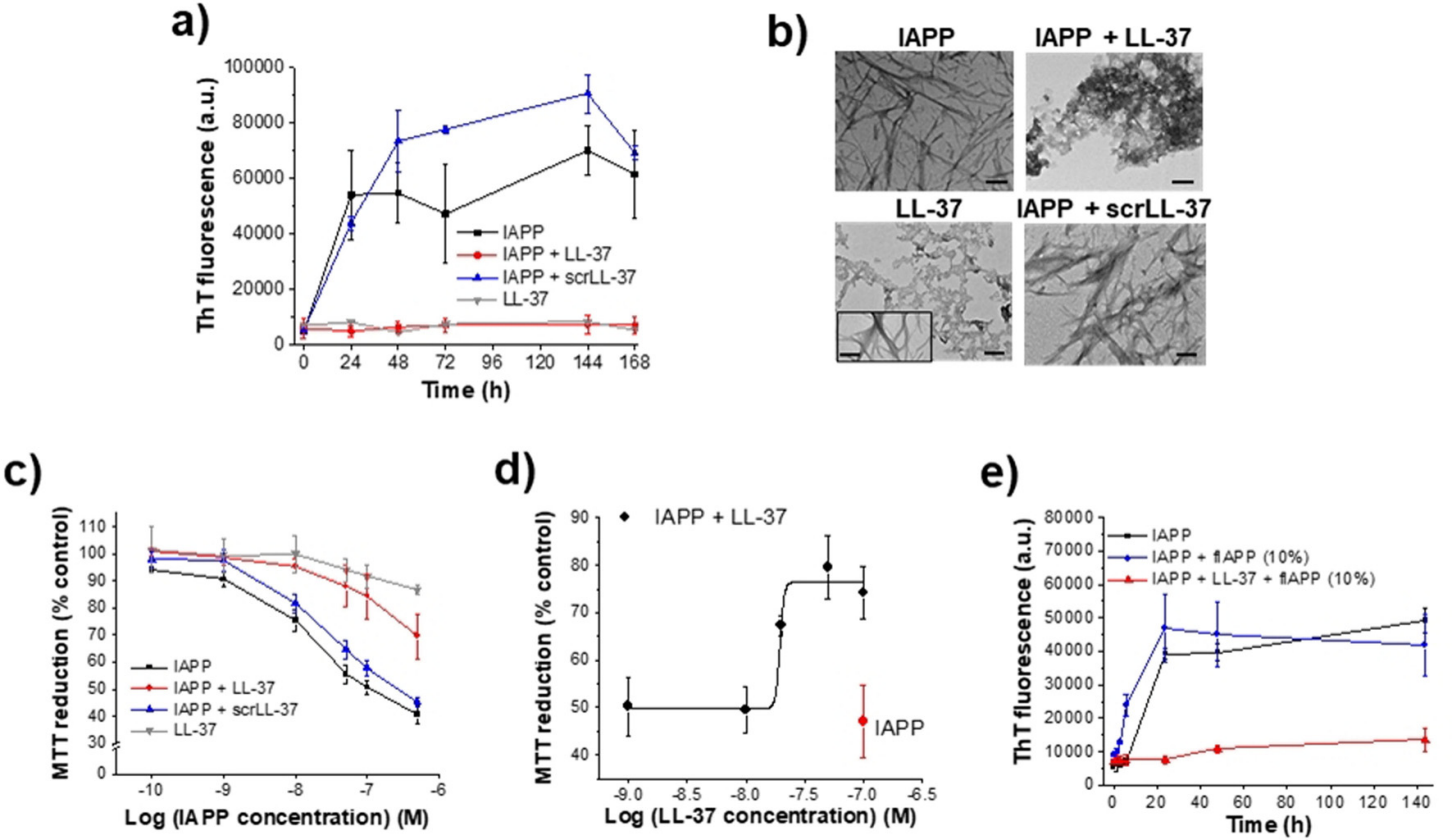
Effects of LL‐37 on IAPP amyloid self‐assembly and cell‐damaging effects. a) Fibrillogenesis of IAPP (16.5 μm) alone or with LL‐37 (1:1) determined by ThT binding (means±SD, 3 assays). LL‐37 alone is shown for comparison (1 assay). b) TEM images of 7 days aged solutions from (a) as indicated (bars, 100 nm); inset in LL‐37 image shows LL‐37 fibrils (minor population). c) Cell viability of cultured RIN5fm cells after treatment with IAPP and its mixtures from 1a (7 days aged) determined by MTT reduction [mean±SD, 3 assays (*n*=3 each)]; effects of LL‐37 alone are also shown (1 assay, *n*=3). d) IC_50_ of inhibitory effect of LL‐37 on IAPP cytotoxicity determined by titration of IAPP (100 nm; red symbol) with LL‐37 and MTT reduction [mean±SD, 3 titration assays (*n*=3 each)]. e) Fibrillogenesis of IAPP (16.5 μm) alone or with LL‐37 (1:1) following seeding with fIAPP (10 %) determined by ThT binding (mean±SD, 3 assays).

To characterize the LL‐37‐IAPP interaction, we performed fluorescence spectroscopic titrations, CD spectroscopy, cross‐linking, and dot blots (DBs). First, titration of N‐terminal fluorescein‐labeled IAPP (Fluos‐IAPP, 5 nm) with various amounts of LL‐37 was performed; its interaction with 100‐fold molar excess of LL‐37 resulted in a 322 % increase in its fluorescence emission (Figure 2 a). The titration yielded an apparent (app.) *K*_d_ of 88.1(±12) nm consistent with a high‐affinity interaction (Figure 2 a). Since freshly made solutions of Fluos‐IAPP at 5 nm consist mainly of monomers, these results suggest that LL‐37 binds monomeric IAPP with nanomolar affinity.6d To find out whether LL‐37 binds IAPP fibrils as well, DBs were performed using N‐terminal fluorescein‐labeled LL‐37 (FAM‐LL‐37). In fact, FAM‐LL‐37 bound both IAPP fibrils and monomers (Figure 2 b).

**Figure 2:**
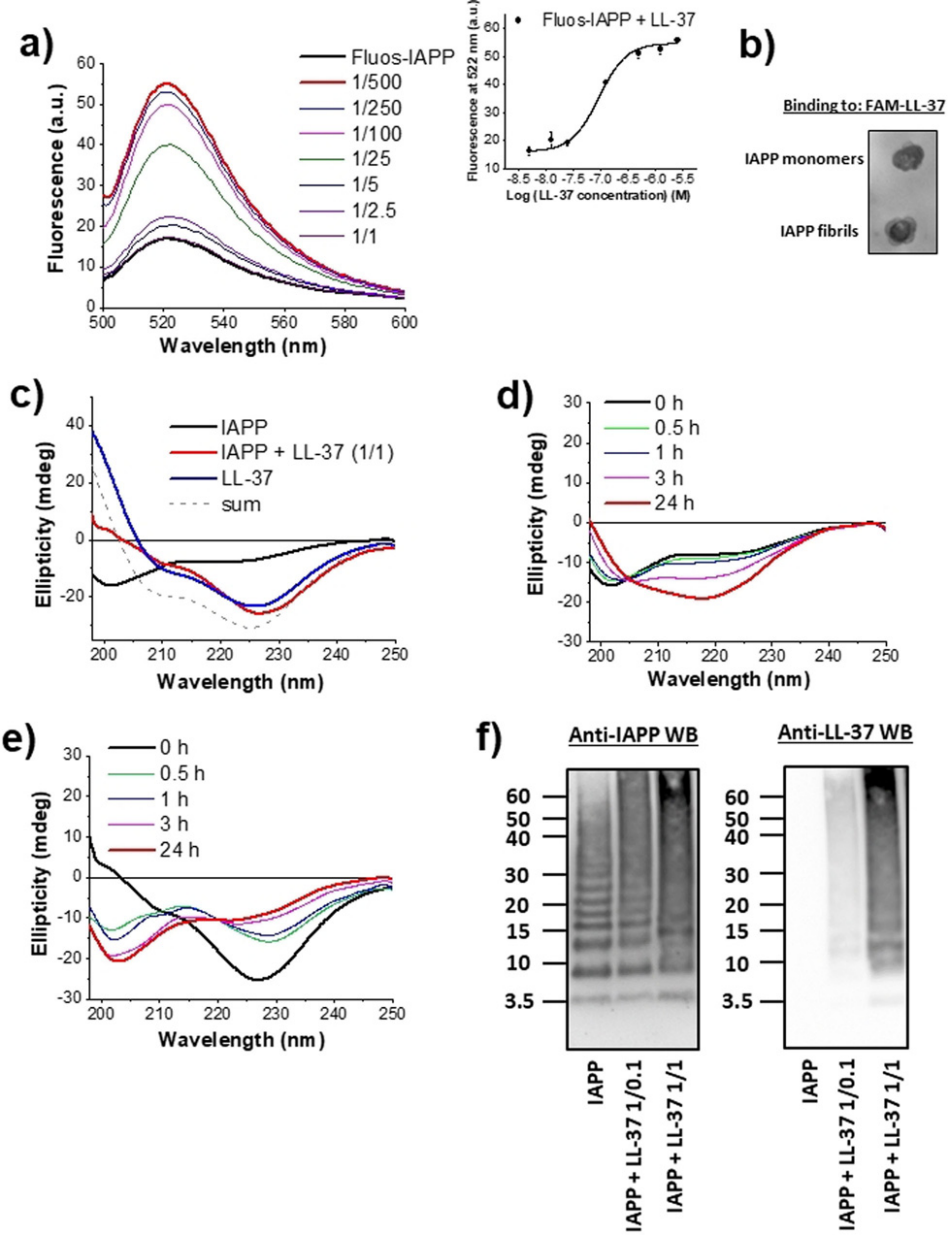
Characterization of the LL‐37‐IAPP interaction. a) Determination of the app. *K*_d_ by fluorescence spectroscopic titrations. Fluorescence emission spectra of Fluos‐IAPP (5 nm) alone or with various amounts of LL‐37 (pH 7.4) as indicated. Inset: binding curve (mean±SD, 3 titration assays). b) Binding of FAM‐LL‐37 to IAPP monomers and fibrils as determined by DB. IAPP monomers and fibrils (40 μg) were spotted on a nitrocellulose membrane and probed with FAM‐LL‐37 (200 nm; results representative of 4 assays). c) Far‐UV CD spectra of IAPP (5 μm), IAPP‐LL‐37 (1:1; 5 μm each), and LL‐37 (5 μm, 0 h,pH 7.4). The sum of the spectra of LL‐37 and IAPP is also shown. d, e) Kinetic follow‐up of IAPP misfolding alone (d) or its 1:1 mixture with LL‐37 (e) through far‐UV CD spectroscopy. Conditions as in (c). f) Characterization of IAPP/LL‐37 hetero‐assemblies through cross‐linking with glutaraldehyde (pH 7.4), NuPAGE, and western blotting (IAPP 30 μm; IAPP/LL‐37 1:0.1 or 1:1). A representative gel (*n*>5) is shown.

To determine the effects of LL‐37 on IAPP conformation and misfolding, far‐UV CD spectra of IAPP, LL‐37, and the IAPP/LL‐37 mixture (1:1) were measured at various incubation time points (Figures 2 c–e).^10^ The spectrum of IAPP (0 h) exhibited a strong minimum at approximately 200 nm, which is indicative of large amounts of unordered structure (Figure 2 c). By contrast, the spectrum of LL‐37 exhibited a strong n→π* minimum at around 227 nm, a smaller one at around 210 nm, and a maximum at around 198 nm. These features were indicative of large amounts of α‐helix and/or β‐sheet/turn structure. Importantly, the spectrum of the mixture differed from the sum of the spectra confirming the interaction (Figure 2 c). Also, the CD spectra of the mixture and of LL‐37 were very similar to each other; α‐helical homo‐ or hetero‐oligomers could account for the 227 and 210 nm minima (Figure 2 c).9b, 11 In fact, LL‐37 has a well‐known propensity to self‐assemble into α‐helical oligomers, while α‐helix‐mediated homo‐dimerization might precede IAPP amyloidogenesis.3d, 10, 11, 12 Of note, scrLL‐37 (1:1 relative to IAPP) did not affect IAPP conformation (Figure S2). The CD spectra of IAPP at various incubation time points indicated a conformational transition into β‐sheet‐rich assemblies, leading to fibril formation and precipitation (24 h; Figure 2 d).^10^ By contrast, the LL‐37/IAPP mixture exhibited a strong time‐dependent increase of random‐coil content and no precipitation occurred (Figure 2 e). Thus, the LL‐37/IAPP interaction yielded soluble, partly disordered hetero‐assemblies that suppressed IAPP fibrillogenesis.

To further characterize the LL‐37/IAPP hetero‐assemblies, cross‐linking studies were performed. IAPP solutions contained low MW oligomers, mostly di‐ to hexamers, and higher MW aggregates (Figure 2 f). A similar pattern was observed in the presence of non‐inhibitory amounts (0.1 equivalents) of LL‐37. By contrast, in the presence of an inhibitory (equimolar) LL‐37 amount, a novel prominent band, which was absent in the IAPP‐only incubations, was found at around 15 kDa and suggested the formation of IAPP/LL‐37 hetero‐tetramers (Figure 2 f). In addition, a strong reduction of low MW oligomeric IAPP bands, likely corresponding to cytotoxic IAPP oligomers, was observed (Figure 2 f). Western blot (WB) with anti‐LL‐37 antibody confirmed the presence of LL‐37 in the 15 kDa band of the IAPP/LL‐37 mixtures (Figure 2 f). Notably, LL‐37 alone also contained a band at around 15 KDa corresponding to LL‐37 homo‐tetramers (Figure S3).9b, 11a Together, these studies identified LL‐37/IAPP hetero‐tetramers as major hetero‐oligomeric populations and suggested that their formation may underlie the inhibitory effect of LL‐37. Furthermore, IAPP seeding studies suggest that binding of LL‐37 to IAPP fibrils converts them into seeding‐incompetent assemblies, thereby providing an additional mechanistic explanation for its potent amyloid inhibitor function (Figure 3 and Supporting Information).

**Figure 3:**
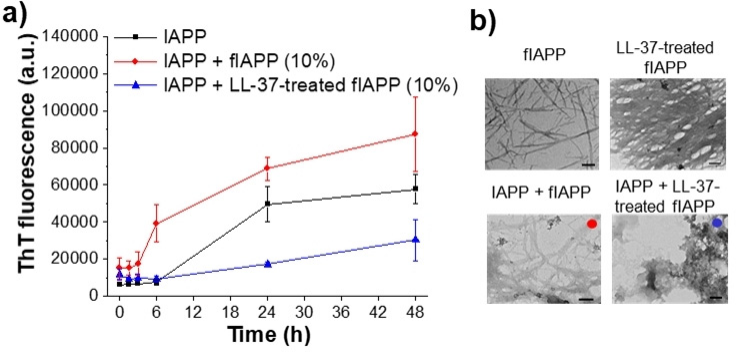
LL‐37 binding to IAPP fibrils (fIAPP) converts them into seeding‐incompetent assemblies: a) Fibrillogenesis of IAPP (16.5 μm) alone or following seeding with 10 % fIAPP or with 10 % LL‐37‐treated fIAPP determined by ThT binding (means±SD, 3 assays). b) TEM images of solutions from (a): fIAPP seeds, LL‐37‐treated fIAPP seeds, and IAPP seeded with fIAPP (10 %, red dot) or LL‐37‐treated fIAPP (10 %, blue dot; both at 6 h). Scale bars=100 nm.

Specific partial LL‐37 sequences within its central/C‐terminal parts such as LL‐37(17(18)‐29) or LL‐37(13‐32) have been found to be sufficient for antibacterial, antiviral, or immunomodulatory activity and are thus being used for drug design.3a, 3c, 3d, 3e, 5 To find out whether the amyloid‐inhibition function of LL‐37 resides within specific sequence parts as well, we dissected it into the two segments: LL‐37(1‐14) and LL‐37(15‐37), which contain the N‐ and central/C‐terminal helical parts, respectively.3c, 13 The peptides were synthesized and their interactions and effects on IAPP amyloid self‐assembly were studied. Importantly, neither segment was able to interfere with IAPP amyloid self‐assembly and cell‐damaging effects (1:1 with IAPP; Figure 4 a,b). In addition, fluorescence titrations revealed that LL‐37(15‐37) bound Fluos‐IAPP with as high affinity (app. *K*_d_=31.9(±2.2) nm) as full length LL‐37; by contrast, a circa 30‐fold weaker binding (app. *K*_d_=2.54(±0.5) μm) was found for LL‐37(1‐14); Figure S4). Thus, while the central/C‐terminal LL‐37 part likely mediates its high‐affinity interaction with IAPP, it is not sufficient for amyloid inhibition; the concerted action of central/C‐terminal and N‐terminal parts appears to be required.

**Figure 4:**
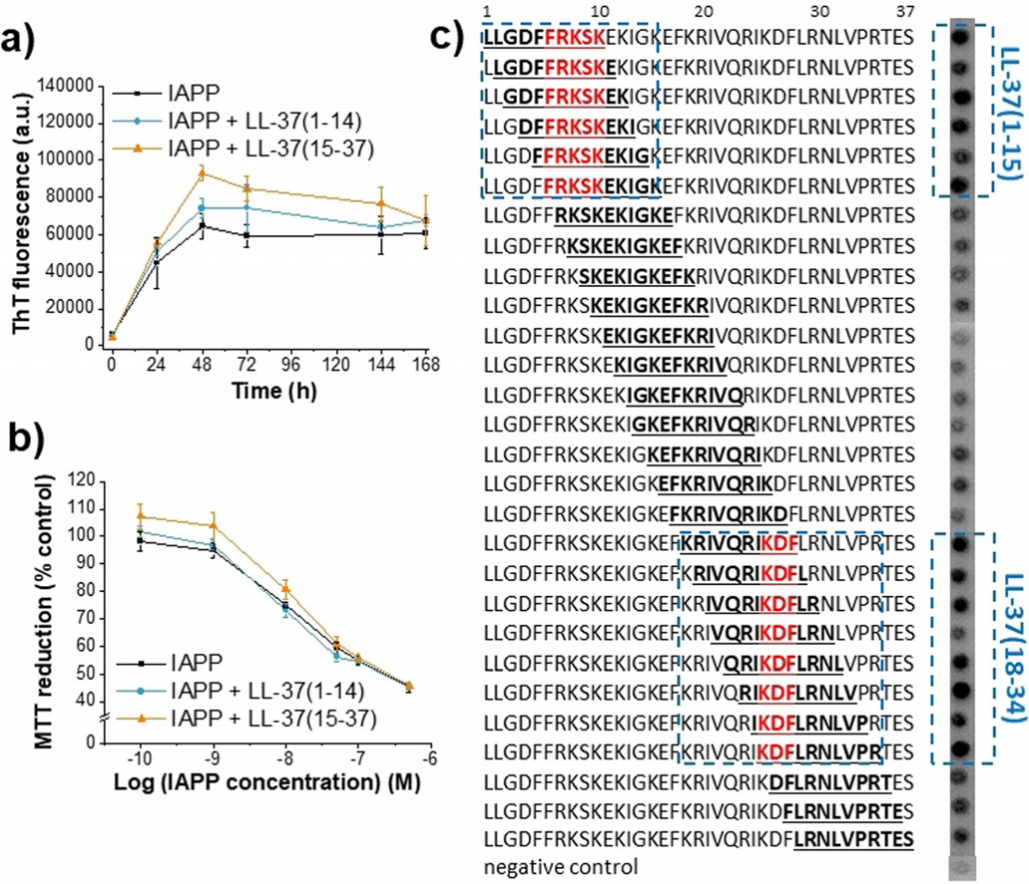
Identification of regions of LL‐37 that mediate its interaction with IAPP and its potent amyloid‐inhibition function. a) Fibrillogenesis of IAPP (16.5 μm) alone or in the presence of LL‐37(1‐14) or LL‐37(15‐37) (1:1) as determined by ThT binding (mean±SD, 3 assays). b) β‐cell‐damaging effects of 24 h aged solutions from (a) determined by MTT reduction [RIN5fm cells; mean±SD, 3 assays (*n*=3 each)]. c) Identification of LL‐37 regions that bind IAPP using peptide microarrays. Glass slides with decamers consisting of overlapping LL‐37 sequences (bold) were incubated with Fluos‐IAPP (1 μm); visualization by fluorescence. Identified IAPP binding clusters are indicated by dashed blue line frames; LL‐37 “binding cores” by red letters (results representative from 4 assays).

To better characterize the LL‐37 regions involved in its interaction with IAPP, we used peptide arrays of LL‐37 decamers covering full‐length LL‐37 and positionally shifted by one residue; peptides were covalently attached on glass slides.^14^ Incubation with Fluos‐IAPP revealed two clusters of 6–8 consecutive IAPP binding segments: the first one in LL‐37(1‐15) and the second one in LL‐37(18‐34) (Figure 4 c). The common sequence parts within each binding cluster, that is, the “binding cores”, were LL‐37(6‐10) or FRKSK at the N‐terminus, and LL‐37(25‐27) or KDF within the C‐terminal part (Figure 4 c). These findings were in line with the LL‐37 dissection studies; in addition, they identified the segments mediating its interaction with IAPP.

In summary, we have identified a high‐affinity interaction between LL‐37 and IAPP that effectively suppresses IAPP amyloid self‐assembly in vitro, along with key LL‐37 segments that mediate this interaction. Our results suggest that the inhibitor function of LL‐37 is mediated by binding to 1) early prefibrillar IAPP species and their sequestration into soluble, non‐fibrillar hetero‐assemblies and 2) IAPP fibrils and their conversion into seeding‐incompetent assemblies. Together with findings by others, our results support the hypothesis that LL‐37 secreted by pancreatic β‐cells or infiltrated neutrophils under conditions of pancreatic inflammation binds IAPP and suppresses its amyloid self‐assembly and related β‐cell damage, thus slowing down T2D pathogenesis (Scheme 2).2a, 2c, 4a Studies on the potential physiological relevance of the LL‐37/IAPP interaction are now of high priority.

**Scheme 2:**
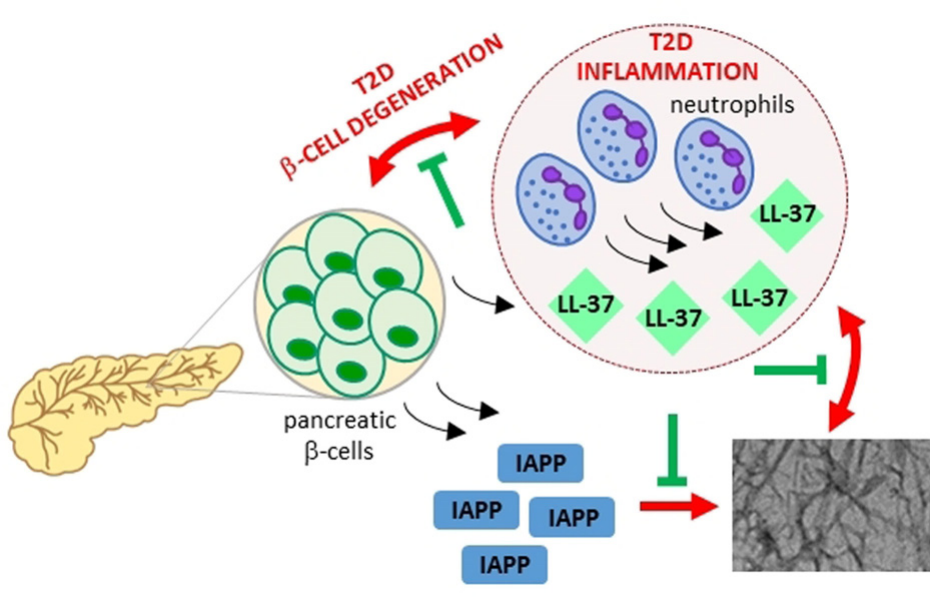
Suggested protective role of the LL‐37/IAPP interaction in pancreatic amyloid formation, inflammation, β‐cell degeneration, and T2D pathogenenesis.

In conclusion, our studies have uncovered a high‐affinity amyloid‐suppressing interaction between a major antimicrobial and immunomodulatory polypeptide and the key amyloid polypeptide of T2D, and offer a molecular basis for the design of novel molecules combining antimicrobial, immunomodulatory, and T2D‐related anti‐amyloid functions as candidates for multifunctional drugs.

## Conflict of interest

Valentina Armiento, Annelise E. Barron, and Aphrodite Kapurniotu are coinventors in a provisional application for a US patent on LL‐37‐based treatment strategies in diabetes.

## Supporting information

As a service to our authors and readers, this journal provides supporting information supplied by the authors. Such materials are peer reviewed and may be re‐organized for online delivery, but are not copy‐edited or typeset. Technical support issues arising from supporting information (other than missing files) should be addressed to the authors.

Supplementary
