# Supplementary material for: The Human Host‐Defense Peptide Cathelicidin LL‐37 is a Nanomolar Inhibitor of Amyloid Self‐Assembly of Islet Amyloid Polypeptide (IAPP)
Source: Angew Chem Int Ed Engl. 2020 Apr 30;59(31):12837–41. doi: 10.1002/anie.202000148 (PMC7497016; doi:10.1002/anie.202000148)
Supplement: Supplementary file 1 — Supplementary [file ANIE-59-12837-s001.pdf]

Angewandte Chemie
Eine Zeitschrift der Gesellschaft Deutscher Chemiker

Supporting Information

The Human Host-Defense Peptide Cathelicidin LL-37 is a Nanomolar Inhibitor of Amyloid Self-Assembly of Islet Amyloid Polypeptide (IAPP)

*Valentina Armiento, Kathleen Hille, Denise Naltsas, Jennifer S. Lin, Annelise E. Barron, and Aphrodite Kapurniotu**

anie_202000148_sm_miscellaneous_information.pdf

Author Contributions

V.A. Formal analysis: Lead; Investigation: Lead; Validation: Lead; Visualization: Equal; Writing - Original Draft: Supporting; Writing - Review & Editing: Supporting
K.H. Investigation: Supporting
D.N. Investigation: Supporting; Methodology: Supporting
J.L. Conceptualization: Supporting; Writing - Review & Editing: Supporting
A.B. Conceptualization: Supporting; Resources: Supporting; Writing - Review & Editing: Supporting
A.K. Conceptualization: Lead; Funding acquisition: Lead; Methodology: Lead; Project administration: Lead; Resources: Lead; Supervision: Lead; Visualization: Equal; Writing - Original Draft: Lead; Writing - Review & Editing: Lead.

Table of Contents

Supporting Text

Studies on the effect of LL-37 on the seeding ability of IAPP fibrils. 2

Experimental part

Peptides and peptide synthesis. 3
Thioflavin T (ThT) binding assays. 3,4
Assessment of cell damage by the MTT reduction assay. 4
Transmission electron microscopy (TEM). 5
Far-UV CD spectroscopy. 5
Fluorescence spectroscopic titrations. 5
Cross-linking, NuPAGE, and Western blot analysis. 5,6
Dot Blot analysis. 6
Determination of LL-37 binding sites by using peptide arrays. 6,7
Sequence alignment using LALIGN. 7

References 7

Supporting Figures

Figure S1. Dose-dependence of the inhibitory effect of LL-37 on IAPP fibrillogenesis. 8
Figure S2. Effects of scrLL-37 on IAPP fibrillogenesis, cell-damaging effects, and conformation. 9
Figure S3. Characterization of LL-37 homo-oligomers via cross-linking with glutaraldehyde, NuPAGE, and WB. 10
Figure S4. Determination of the app. K_ds of IAPP interactions with the partial LL-37 segments LL-37(1-14) and LL(15-37) by fluorescence spectroscopic titrations. 11

Supporting Text

Studies on the effect of LL-37 on the seeding ability of IAPP fibrils

To address the question whether binding of LL-37 to IAPP fibrils (fIAPP) might contribute to its inhibitory effect on IAPP amyloid self-assembly, preformed fIAPP versus LL-37-treated fIAPP (i.e. fIAPP incubated with LL-37 (10-fold) for 24 h) were studied regarding their ability to act as seeds of IAPP fibrillogenesis (Figure 3). In contrast to untreated fIAPP (10%), which strongly accelerated IAPP fibrillogenesis, LL-37-treated fIAPP (10%) were unable to do so (Figure 3). TEM revealed marked morphological differences between fIAPP and LL-37-treated fIAPP, which stick laterally to each other into large sheet-like assemblies (Figure 3b). These results suggested that binding of LL-37 to IAPP fibrils converts them into seeding incompetent assemblies, providing thus an additional mechanistic explanation for its potent amyloid inhibitor function.

Experimental part

Materials and methods

Peptides and peptide synthesis

IAPP was synthesized using Fmoc-solid phase synthesis strategy on Rink resin, oxidized with air and purified with reverse phase (RP) HPLC as previously described.[1] IAPP stock solutions were made by dissolving the peptide in 1,1,3,3,3,3-hexafluoro-2-isopropanol (HFIP) (at 4°C) and filtering the solution as described; IAPP concentration was determined by UV spectroscopy.[2] N^{α}-amino-terminal fluorescein labeled IAPP (Fluos-IAPP) was synthesized, purified (MALDI-TOF MS: found MH^+, 4261.2; calculated 4262.2), and handled as previously described.[1, 3] LL-37 was purchased from BACHEM and from AnaSpec; its stock solutions were made by dissolving it in HFIP (at 4°C); its concentration was determined by its weight and the BCA assay. Scrambled LL-37 (scrLL-37) and N-amino-terminal fluorescein labeled LL-37 (FAM-LL-37) were purchased from Anaspec; of note, a 6-aminohexanoic acid spacer was included between the fluorescein (FAM) moiety and the N-terminus of LL-37 in FAM-LL-37. Synthetic glucagon (control for dot blot assays) was from BACHEM. The LL-37 partial segments LL-37(1-14) and LL-37(15-37) were synthesized on Wang-resin using previously established Fmoc-SPPS protocols both manually and by a CS336X peptide synthesizer (CS Bio).[4] Briefly, couplings were performed (twice or 3 times) using standard Fmoc-protected amino acids (3-fold molar excess) and as coupling reagents *N,N,N′,N′*-tetramethyl-*O*-(1*H*-benzotriazol-1-yl)uronium hexafluorophosphate (HBTU) or 2-(7-aza-1H-benzotriazole-1-yl)-1,1,3,3-tetramethyluronium hexafluorophosphate (HATU) (3-fold molar excess) for selected couplings, and N,N-diisopropylethylamine (DIEA) (4.5 molar excess) in N,N-dimethylformamide (DMF). In the case of LL-37(1-14), most couplings were performed twice using HBTU as coupling agent except for Lys^{10}, Glu^{11}, and Lys^{12} for which HATU was used for the first coupling; in addition, 3 couplings were performed for Phe^{5}, Phe^{6}, and Arg^{7} using HATU for the first two of them. In the case of LL-37(15-37), most couplings were repeated three times using HATU for the first one and HBTU for the following ones; double couplings were performed only within LL-37(15-19), and for residues Lys^{25}, Asp^{26}, Pro^{33}, and Arg^{34}. Cleavages of the two LL-37 segments from the resin were carried out by trifluoroacetic acid/water (95/5, v/v) (3 h).[4c] Their RP-HPLC purifications were performed using a Nucleosil 100 C18 (250 mm x 8 mm; particle size, 7 µm) column as described.[4c] Peptide purity (including the purity of commercially obtained LL-37, scrLL-37 and FAM-LL-37) was verified by MALDI-TOF MS. In the case of LL-37(1-14): found MH^+, 1638.3 (calculated, 1638.9); in the case of LL-37(15-37), found MH^+, 2873.9 (calculated, 2873.6). Stocks of LL-37(1-14) and LL-37(15-37) were made in HFIP (4°C) and their concentrations were determined by their weight and confirmed by the BCA assay.

Thioflavin T (ThT) binding assays

The effects of LL-37 and the other peptides on kinetics of IAPP fibrillogenesis were investigated by using the thioflavin T (ThT) binding assay using a previously established protocol.[5] Briefly, IAPP alone (16.5 µM) and its mixtures with LL-37 and the other peptides were incubated in ThT

assay buffer (aqueous 50 mM sodium phosphate buffer, pH 7.4, containing 100 mM NaCl and 0.5% HFIP) at the indicated molar ratios (20°C) (non-stirring conditions).[5] Of note, an incubation of LL-37 alone was also included.[6] At the indicated time points aliquots were mixed with the ThT solution consisting of 20 µM ThT in 0.05 M glycine/NaOH (pH 8.5) and binding was determined by measuring fluorescence emission at 486 nm upon excitation at 450 nm using a Multilabel reader VictorX3 (Perkin Elmer Life Sciences).[5] For studying the effect of LL-37 on nucleation of IAPP fibrillogenesis, i.e. following seeding with preformed IAPP fibrils (fIAPP) (10%), incubations of IAPP (16.5 µM) and its mixtures with LL-37 (1/1) were performed as above at room temperature. An aliquot of a solution consisting mostly of IAPP fibrils (fIAPP) based on ThT binding and TEM (Fig. 1a,b) (7 days aged IAPP (16.5 µM) made as above) was added to the incubations resulting in a final seed (fIAPP) concentration of 1.65 µM. Incubations of (unseeded) IAPP alone (16.5 µM) and of 10% fIAPP alone were included as controls. ThT binding was determined at the indicated time points as above. To study the effect of LL-37-treated IAPP fibrils (LL-37-treated fIAPP) on kinetics of IAPP fibrillogenesis in comparison to the seeding effect of fIAPP, an aliquot of a solution consisting mostly of fIAPP (16.5 µM fIAPP, 7 days aged; see above) was added to solid LL-37 (10-fold molar excess) and the mixture was incubated for 24 h yielding "LL-37-treated fIAPP" (Fig. 3b). Of note, binding of LL-37 to fIAPP was confirmed by a dot blot (DB) assay (Figure 2b and data not shown); in addition, a DB assay was also applied to confirm that the same amounts of fIAPP were present in the aliquots of fIAPP and the LL-37-treated fIAPP used for the seeding assays (data not shown). An aliquot of the fIAPP containing solution (treated in the same way as the LL-37-treated aliquot but w/o LL-37) was used to determine the seeding effect of fIAPP. Solutions containing (unseeded) IAPP alone (16.5 µM), IAPP seeded with fIAPP (10%), and IAPP seeded with LL-37-treated fIAPP (10%) were made in ThT assay buffer and kinetics of fibrillogenesis were determined by the ThT binding assay as described above. Of note, very similar results as the ones shown in Fig. 3a were obtained when LL-37-treated fIAPP were separated from unbound LL-37 by centrifugation prior to their use in the IAPP seeding assay (data not shown).

Assessment of cell damage by the MTT reduction assay

Effects of LL-37 and the other peptides on formation of β-cell damaging IAPP assemblies were studied in the rat insulinoma cell line RIN5fm using the peptide solutions applied for the ThT binding assays as previously described.[1, 5] Briefly, RIN5fm cells were cultured and plated in 96-well plates as described.[4a] Solutions of IAPP alone and its mixtures with peptides were aged in ThT assay buffer as described under "ThT binding assays". At the indicated incubation time points (24 h or 7 days) aliquots were diluted with cell culture medium and added to the cells. Following incubation with the cells for ~20 h (37°C, humified atmosphere containing 5% CO_2), cell damage was verified by the MTT reduction assay.[1, 4a, 5b] For the determination of the IC_{50} of the inhibitory effect of LL-37 on formation of cytotoxic IAPP aggregates, 24 h aged IAPP (100 nM) alone and its mixtures with various amounts of LL-37 (made as under ThT binding assay) were added to the cells and cell viability was determined by the MTT reduction assay as above.[5-6]

Transmission Electron Microscopy (TEM)

TEM samples were prepared applying 10 µl aliquots of the solutions used in the ThT binding and MTT assays on carbon-coated grids at the indicated time points. The grids were washed using ddH_2O and stained with aqueous 2% (w/v) uranyl acetate solution.[1-2, 5b] Examination of the grids was done with a JEOL 1400 Plus electron microscope at 120 kV.

Far-UV CD Spectroscopy

Far-UV CD studies were performed using a Jasco 715 spectropolarimeter. Spectra were recorded at room temperature between 195 and 250 nm, at 0.1 nm intervals, and with a response time of 1 second. Each spectrum is an average of 3 spectra. All CD studies were performed in aqueous 10 mM sodium phosphate buffer (pH 7.4) containing 1% HFIP (CD assay buffer) at room temperature; this assay system has been earlier developed and found to be suitable for following kinetics of IAPP (5 µM) misfolding into β-sheets and amyloid fibrils alone or in the presence of inhibitors.[1-3, 4b, 4d, 5] Briefly, peptide stocks in HFIP were freshly made (4°C), diluted with assay buffer (room temperature) at the indicated concentrations within the cuvette, and following gentle mixing spectra were measured immediately or at the indicated incubation time points. For the studies addressing the interactions between LL-37 or scrLL-37 and IAPP, peptide mixtures (1/1) were prepared in HFIP (4°C) and diluted with assay buffer in the cuvette at the indicated concentrations (5 µM each) (room temperature); CD spectra were measured as above. Of note, CD studies on IAPP alone and LL-37 or scrLL-37 alone (from the same stocks; 5 µM) were also performed in parallel. The CD spectrum of IAPP at the incubation time point of 24 h (endpoint) was measured after gentle mixing to redissolve precipitated aggregates. The CD spectrum of the buffer was always subtracted from the CD spectra of the peptide solutions.

Fluorescence spectroscopic titrations

Fluorescence spectroscopic titration studies were performed with a JASCO FP-6500 fluorescence spectrophotometer using a previously described experimental protocol.[1, 5, 7] Briefly, excitation was at 492 nm and emission spectra were recorded between 500 and 600 nm. The apparent (app.) K_ds of the interactions of IAPP with LL-37 and its segments LL-37(1-14) and LL-37(15-37) were quantified by titrating synthetic N^{α}-amino-terminal fluorescein labeled IAPP (5 nM) with various amounts of each of the peptides. For all experiments, freshly made stocks of peptides and their fluorescently labeled analogs in HFIP were used. Measurements were performed in 10 mM sodium phosphate buffer (pH 7.4) containing 1% HFIP within 2-5 min following solution preparation at room temperature. Of note, under these conditions freshly made Fluos-IAPP (5 nM) solutions consist mostly of monomers.[1] App. K_ds were calculated using 1/1 binding models as previously described and are means (± SD) of three binding curves.[1, 3, 5, 7]

Cross-linking, NuPAGE, and Western Blot analysis

Cross-linking studies were preformed using a previously developed assay system.[5] Briefly, solutions of IAPP alone (30 µM) and its mixtures with LL-37 or scrLL-37 at the indicated molar ratios (IAPP/LL-37 at 1/1 or 1/0.1) were prepared in aqueous sodium phosphate buffer, pH 7.4,

and incubated for 30 min at room temperature; of note, incubations of LL-37 alone (at the same concentrations as in its mixtures with IAPP) were made as well. Solutions were cross-linked using 25% aqueous glutaraldehyde (Sigma-Aldrich) and 10% aqueous trichloroacetic acid (TCA) was used to precipitate cross-linked peptides.[1, 7] Pellets were dissolved in reducing NuPAGE sample buffer, boiled for 5 min, and subjected to NuPAGE electrophoresis in 4-12% Bis-Tris gels with MES running buffer (Invitrogen). The same volume of each solution (same IAPP amount) was loaded in all lanes. Peptides were blotted using a XCell II Blot Module blotting system (Invitrogen). IAPP or LL-37 were detected using a polyclonal rabbit anti-IAPP antibody (Peninsula) or a monoclonal mouse anti-LL-37 antibody (Santa Cruz Biotechnology), respectively in combination with suitable peroxidase (POD)-coupled secondary antibodies (Pierce & Amersham) and the Super Signal West Dura Extended Duration Substrate (Pierce). Of note, previous studies provided evidence for the specificity of the cross-linking assay; in addition, no new bands were observed in IAPP-scrLL-37 (1/1) mixtures (data not shown).[5]

Dot blot analysis

IAPP monomers or IAPP fibrils (fIAPP) containing solutions (different amounts up to 40 µg) were spotted onto a nitrocellulose membrane. These solutions were prepared by incubating an IAPP solution (1 mg/ml) in ThT assay buffer for 0 h (“monomers”) or 24 h (“fibrils”); the presence of fibrils was confirmed by ThT binding and TEM (not shown). The membrane was washed with TBSn (20 mM Tris/HCl, 150 mM NaCl and 0.05% Tween-20), blocked with 5% milk in TBSn overnight at 10°C, and washed again with TBSn. Then, the membrane was incubated with N-terminal fluorescein labeled LL-37 (FAM-LL-37 from AnaSpec; see under “Peptides and peptide synthesis”) (200 nM) in ThT assay buffer containing 1% HFIP overnight at 10°C. Following washings with incubation buffer and TBSn, bound FAM-LL-37 was visualized with a LAS-4000mini instrument (Fujifilm). Of note, glucagon fibrils were spotted as well to control for the specificity of the observed strong binding of FAM-LL-37 to fIAPP (not shown). Glucagon fibrils were made by incubating glucagon in 10 mM HCl (2 µg/µl) (10 days) followed by neutralization with 10 mM NaOH; ThT binding and TEM confirmed fibril formation (not shown). The ThT buffer alone was also spotted to control for NSB. In addition, to control for the interference of fibril autofluorescence, a membrane containing spotted fIAPP which had been incubated in buffer alone w/o FAM-LL-37 was included in each assay; in general, fIAPP autofluorescence contributed up to 25% of the total amount of fluorescence observed in fIAPP bound to FAM-LL-37.

Determination of LL-37 binding sites by using peptide arrays

A peptide array consisting of LL-37 decamers covering the full length LL-37 sequence and positionally shifted by one residue was synthesized on a modified cellulose membrane support using stepwise SPOT synthesis protocols and a MultiPep RSi (Intavis) peptide synthesizer.[7-8] Thereafter, peptides were immobilized on a glass slide according to the manufacturer’s instructions followed by a blocking step using 1% BSA in TBSn for 4 h (room temperature). The glass slide with the peptide array was incubated with a solution of Fluos-IAPP (1 µM in TBSn containing 1% BSA) for ~12 h at 10°C followed by washing with TBSn. Visualization of bound Fluos-IAPP was

performed with a LAS-4000mini instrument (Fujifilm).

Sequence alignment using LALIGN

The sequence alignment of IAPP and LL-37 was done with the program LALIGN (Author: Bill Pearson; https://embnet.vital-it.ch/software/LALIGN_form.html). Of note, this program was previously used for the comparison of the Aβ and IAPP sequences to each other.[9] A global alignment method was used with 3 reported sub-alignments; E-value threshold was set to 10.0, the scoring matrix used is BLOSUM50, opening gap penalty was set to -12 and extending gap penalty to -2 (default values). The LALIGN program implements the algorithm of Huang and Miller.[10]

References

[1] L. M. Yan, M. Tatarek-Nossol, A. Velkova, A. Kazantzis, A. Kapurniotu, *Proc Natl Acad Sci U S A* **2006**, *103*, 2046-2051.

[2] R. Kayed, J. Bernhagen, N. Greenfield, K. Sweimeh, H. Brunner, W. Voelter, A. Kapurniotu, *J Mol Biol* **1999**, *287*, 781-796.

[3] M. Tatarek-Nossol, L. M. Yan, A. Schmauder, K. Tenidis, G. Westermark, A. Kapurniotu, *Chem Biol* **2005**, *12*, 797-809.

[4] aK. Tenidis, M. Waldner, J. Bernhagen, W. Fischle, M. Bergmann, M. Weber, M.-L. Merkle, W. Voelter, H. Brunner, A. Kapurniotu, *J Mol Biol* **2000**, *295*, 1055-1071; bA. Kapurniotu, A. Schmauder, K. Tenidis, *J Mol Biol* **2002**, *315*, 339-350; cA. Kazantzis, M. Waldner, J. W. Taylor, A. Kapurniotu, *Eur. J. Biochem.* **2002**, *269*, 780-791; dM. Bakou, K. Hille, M. Kracklauer, A. Spanopoulou, C. V. Frost, E. Malideli, L. M. Yan, A. Caporale, M. Zacharias, A. Kapurniotu, *J Biol Chem* **2017**, *292*, 14587-14602.

[5] aE. Andreetto, E. Malideli, L. M. Yan, M. Kracklauer, K. Farbiarz, M. Tatarek-Nossol, G. Rammes, E. Prade, T. Neumuller, A. Caporale, A. Spanopoulou, M. Bakou, B. Reif, A. Kapurniotu, *Angew Chem Int Ed Engl* **2015**, *54*, 13095-13100; bL. M. Yan, A. Velkova, M. Tatarek-Nossol, E. Andreetto, A. Kapurniotu, *Angew Chem Int Ed Engl* **2007**, *46*, 1246-1252.

[6] E. Sancho-Vaello, P. Francois, E. J. Bonetti, H. Lilie, S. Finger, F. Gil-Ortiz, D. Gil-Carton, K. Zeth, *Sci Rep* **2017**, *7*, 15371.

[7] E. Andreetto, L. M. Yan, M. Tatarek-Nossol, A. Velkova, R. Frank, A. Kapurniotu, *Angew Chem Int Ed Engl* **2010**, *49*, 3081-3085.

[8] aC. Katz, L. Levy-Beladev, S. Rotem-Bamberger, T. Rito, S. G. Rudiger, A. Friedler, *Chem Soc Rev* **2011**, *40*, 2131-2145; bM. Lacy, C. Kontos, M. Brandhofer, K. Hille, S. Groning, D. Sinitski, P. Bourilhon, E. Rosenberg, C. Krammer, T. Thavayogarajah, G. Pantouris, M. Bakou, C. Weber, E. Lolis, J. Bernhagen, A. Kapurniotu, *Sci Rep* **2018**, *8*, 5171.

[9] B. O'Nuallain, A. D. Williams, P. Westermark, R. Wetzel, *J Biol Chem* **2004**, *279*, 17490-17499.

[10] X. Huang, W. Miller, *Advs. Appl. Mathem.* **1991**, *12*, 337-357.

Supporting Figures

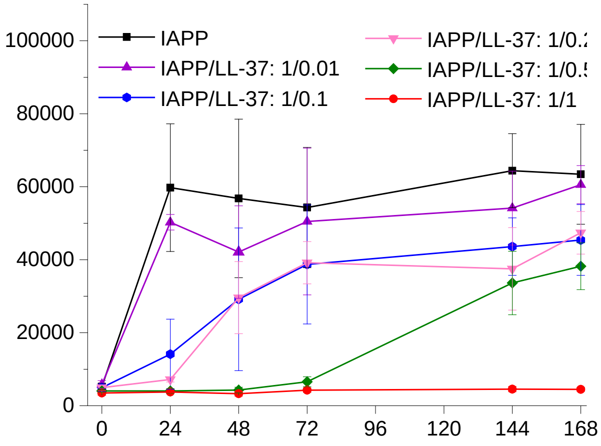

Figure S1. Dose-dependence of the inhibitory effect of LL-37 on IAPP fibrillogenesis: Fibrillogenesis of IAPP (16.5 μM) alone or with different molar ratios of LL-37 as indicated was determined by the ThT binding assay (means (±SD), 3 assays).

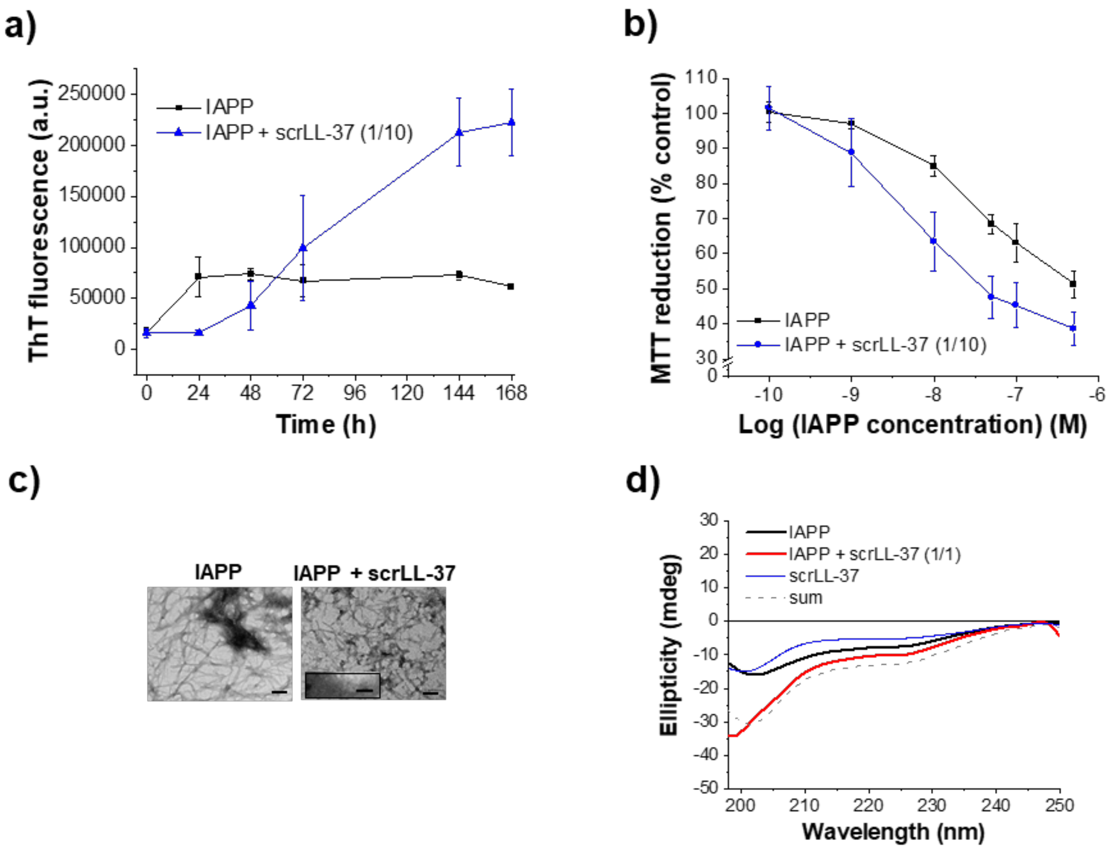

Figure S2. Effects of scrLL-37 on IAPP fibrillogenesis, cell-damaging effects, and conformation: a) Fibrillogenesis of IAPP (16.5 µM) alone or with scrLL-37 (IAPP/scrLL-37, 1/10) determined by the ThT binding assay (means (±SD), 3 assays). b) Effects on IAPP cytotoxicity: Solutions from S2a (7 days aged) were added to RIN5fm cells and cell damage was assessed by MTT reduction (means (±SD), 3 assays (n=3 each)). c) TEM images of solutions (7 days aged) from S2a of IAPP alone and its mixture with scrLL-37; bars, 100 nm). In the inset of the TEM image of the mixture, amorphous aggregates, found to be in addition to the fibrils a major aggregate population and most likely corresponding to scrLL-37 (10-fold excess), are shown. d) Effects of scrLL-37 on IAPP conformation studied by far-UV CD spectroscopy: CD spectra of IAPP (5 µM), the mixture of IAPP with scrLL-37 (1/1; 5 µM each), and scrLL-37 (5 µM) (pH 7.4) are shown; for comparison, the sum of the spectra of scrLL-37 and IAPP is also shown.

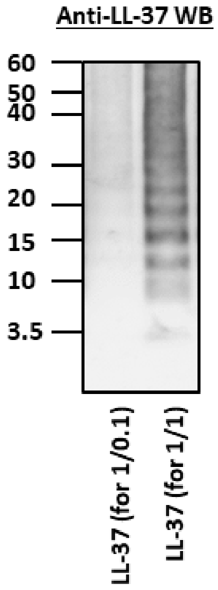

Figure S3. Characterization of LL-37 homo-oligomers (including homo-tetramers at ~15 kDa) via cross-linking with glutaraldehyde, NuPAGE, and WB with anti-LL-37 antibody: LL-37 was incubated (30 min) at concentrations of 3 and 30 µM corresponding to IAPP/LL-37 ratios of 1/0.1 or 1/1 alone or in the presence of IAPP (30 µM; gel shown in Fig. 2f) (pH 7.4). The blot shown is representative of 3 experiments.

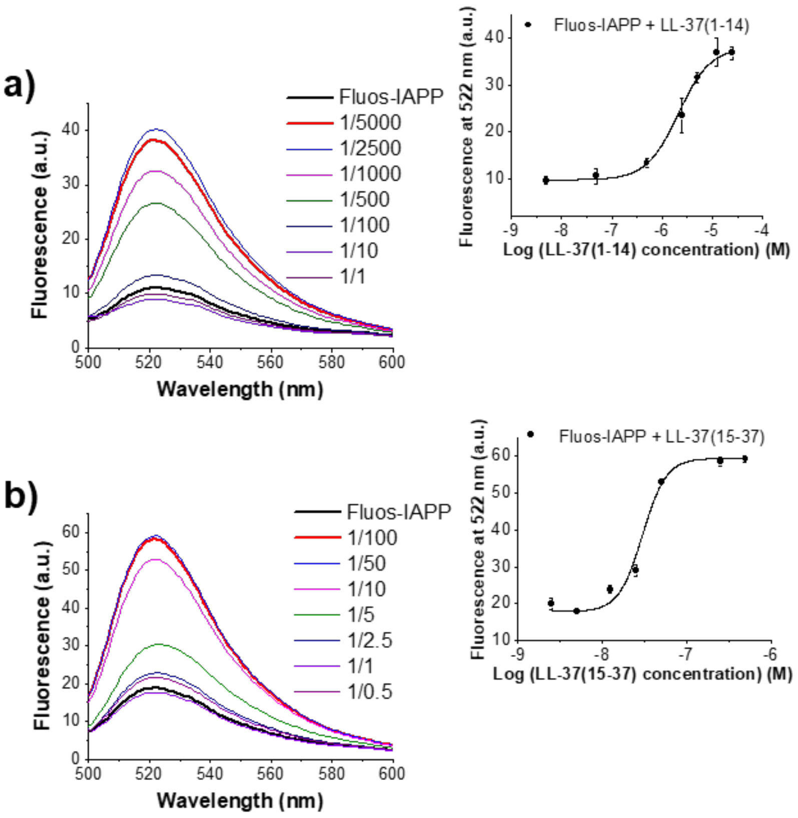

Figure S4. Determination of the app. K_ds of the IAPP interaction with the partial LL-37 segments LL-37(1-14) (a) and LL-37(15-37) (b) by fluorescence spectroscopic titrations. Spectra of Fluos-IAPP (5 nM) alone and after titration with various amounts of LL-37(1-14) (a) or LL-37(15-37) (b) as indicated. Insets, binding curves (means (±SD), 3 titration assays); determined app. K_ds: 2.54 (±0.5) µM for LL-37(1-14) and 31.9 (±2.2) nM for LL-37(15-37).
